# Synergistic Interaction of Histone Deacetylase 6- and MEK-Inhibitors in Castration-Resistant Prostate Cancer Cells

**DOI:** 10.3389/fcell.2020.00610

**Published:** 2020-07-10

**Authors:** Cristina Corno, Noemi Arrighetti, Emilio Ciusani, Elisabetta Corna, Nives Carenini, Nadia Zaffaroni, Laura Gatti, Paola Perego

**Affiliations:** ^1^Molecular Pharmacology Unit, Fondazione IRCCS Istituto Nazionale dei Tumori, Milan, Italy; ^2^Neurological Biochemistry and Neuropharmacology, Fondazione IRCCS Istituto Neurologico Carlo Besta, Milan, Italy; ^3^Cellular Neurobiology Laboratory, Cerebrovascular Unit, Fondazione IRCCS Istituto Neurologico Carlo Besta, Milan, Italy

**Keywords:** prostate cancer, castration-resistance, ricolinostat, selumetinib, paclitaxel

## Abstract

In spite of new knowledge on prostate cancer molecular landscape, this has been only partially translated to the therapeutic setting. The activation of Ras/Mitogen-activated protein kinase (MAPK) signaling plays an important role in progression of prostate cancer in which deregulation of histone deacetylases (HDAC) is frequent. Based on the notion that HDAC inhibitors may reactivate the expression of genes favoring cell response to drugs, the aim of this study was to investigate the interaction between the HDAC6-specific inhibitor ricolinostat (ACY1215) and the MEK-inhibitor selumetinib (AZD6244) to identify effective combinations in prostate cancer models. Using cell lines exhibiting differential activation of survival pathways (PC3, DU145, 22Rv1) and following different treatment schedules, a synergistic interaction was observed in all cell models, the drug combination being particularly effective in 22Rv1 cells. Marginal levels of apoptosis were observed in PC3 cells after combined treatment, whereas higher levels were achieved in DU145 and 22Rv1 cells. RNAi-mediated knockdown of HDAC6 in selumetinib-treated 22Rv1 cells resulted in increased apoptosis. Combined treatment suppressed the constitutively deregulated survival pathways in all cell lines. A decrease of androgen receptor (AR)-dependent gene (KLK2, DUSP1) mRNA levels was observed in 22Rv1 treated cells, associated with increased AR cytoplasmatic expression, suggesting AR signaling down-regulation, not involving Hsp90 acetylation. When a taxane was used in combination with AZD6244 and ACY1215 by a simultaneous schedule, a synergistic cytotoxic effect together with increased apoptosis was evidenced in all cell models. These results support a rational use of targeted agents to improve prostate cancer cell apoptotic response.

## Introduction

Prostate cancer is the second most frequently diagnosed cancer and the third most common cause of cancer-related death in men in Western countries (Siegel et al., 2018). Androgen deprivation by antagonists is an important therapeutic strategy for patients with advanced stage disease (Crawford et al., 2019), but most patients suffer from relapse within a few years, due to the development of a castration-resistant tumor. Treatments for metastatic castration-resistant prostate cancer include secondary hormone therapy (Parker and Sartor, 2011; Scher et al., 2011; Ryan et al., 2013; Beer and Tombal, 2014), immunotherapy (Kantoff et al., 2010), radiopharmaceuticals (Parker et al., 2013), and chemotherapy. Two taxanes, docetaxel and cabazitaxel are now clinical standard treatments (Petrylak et al., 2004; de Bono et al., 2010).

In tumor cells, extracellular signals are transmitted through a network of proteins and inhibition of a single component of a canonical pathway is usually insufficient to produce dramatic effects on cancer cell growth (Natarajan et al., 2006). The activation of the Ras/Mitogen-Activated Protein Kinase (MAPK) signaling pathway plays an important role in progression of prostate cancer to advanced, castration-resistant disease (Bakin et al., 2003). The activation of MAPK, an effector of Ras activation, has been associated with prostate cancer progression (Gioeli et al., 1999). Thus, inhibition of Ras effectors such as MEK could be an effective therapy for advanced prostate cancer (Cossa et al., 2013). A well established set of alterations that could activate MAPK signaling has been identified in prostate cancer and include PTEN loss and, less frequently, BRAF and RAF1 rearrangements (Yap et al., 2016).

Epigenetic modifications which usually occur at an early stage in prostate cancer development play a key role in the patho-physiology of prostate cancer (Cimadamore et al., 2017). Aberrant genomic distribution and global level of histone modifications may lead to silencing of tumor suppressor genes during malignant transformation of prostate cells (Chen et al., 2010). Histone deacetylases (HDAC) have been implicated in prostate cancer progression, providing the rationale for pharmacological treatment of the disease with HDAC inhibitors (HDACi) (Abbas and Gupta, 2008). HDAC isoforms show a variable expression profile in prostate cancer cells, thereby their response to HDACi is not uniform, but cell line-, target- and inhibitor-specific (Waltregny et al., 2004; Kortenhorst et al., 2013). HDAC6 is a Hsp90-deacetylase and an essential positive regulator of its function. Treatment with HDAC6-inhibitors induces hyperacetylation and inhibits ATP-binding and chaperone-function of Hsp90, resulting in polyubiquitination and proteasomal degradation of HSP90-client proteins, including HDAC6 itself, p53 and androgen receptor (AR) (Ai et al., 2009). Thus, HDAC6 appears to be a promising target for castration-resistant prostate carcinoma treatment. Selective HDAC6-inhibitors modulating Hsp90 activity have been proposed for reducing prostate cancer aggressiveness (Seidel et al., 2016).

Based on this background, co-targeting different key players in tumor cell survival (i.e., MAPKs and HDACs) may be effective in inhibiting prostate cancer cell proliferation. In the present study, we examined the efficacy of targeted agent combinations in castration-resistant prostate carcinoma cell lines (DU145 and PC3) characterized by a variable pattern of survival pathway activation, a differential p53 mutational status as well as a different susceptibility to apoptosis. The 22Rv1 cell line, exhibiting both androgen-responsive and androgen-insensitive features (Gregory et al., 1998; Tassinari et al., 2018) was also included in our study. Specifically, we investigated the interaction between the HDAC6-specific inhibitor ricolinostat (ACY1215) and the MEK-inhibitor selumetinib (AZD6244) and cell response to the combination of these agents, using biochemical and molecular approaches.

## Materials and Methods

### Cell Lines and Cell Sensitivity to Drugs

The human castration-resistant prostate carcinoma cell lines DU145, PC3 and 22-Rv1 cells were used in this study (Perego et al., 2006; Tassinari et al., 2018). 22Rv1 cells express the full-length and constitutively active AR variants (Tassinari et al., 2018). All cell lines were cultured in RPMI-1640 medium (Lonza, Milan, Italy) supplemented with 10% fetal bovine serum (Thermo Fisher, Monza, Italy). The cell sensitivity to drugs was measured by a growth-inhibition assay based on cell counting. Exponentially growing cells were seeded in duplicate into six-well plates and, 24 h later, exposed to drugs for 72 h. For combination studies between AZD6244 and ACY1215, the cells were treated according to different schedules: a) 72 h concomitant exposure; b) ACY1215 24 h pre-treatment, followed by 48 h AZD6244 co-exposure. For triple combination studies between AZD6244, ACY1215 and paclitaxel (PTX), the cells were simultaneously treated for 72 h. At the end of treatment, cells were harvested and counted with a cell counter (Coulter Electronics, Luton, United Kingdom). IC_50_ is defined as the drug concentration producing 50% inhibition of cell growth as compared with control. At least 3 independent experiments were performed for each drug or type of treatment. The effect of the combination was evaluated using the Chou and Talalay method (Chou and Talalay, 1984) (Calcusyn software, Biosoft, Cambridge, United Kingdom) in which a combination index (CI) lower than 1 indicates synergism. ACY1215 (Ricolinostat, Rocilinostat, Selleck, Aurogene, Rome, Italy), AZD6244 (Selumetinib, Selleck) and PTX were dissolved in dimethylsulfoxide and diluted in water.

### Apoptosis Analysis

Exponentially growing cells were seeded in 25 cm^2^ flasks and, 24 h later, they were exposed to different concentrations of ACY1215, AZD6244, paclitaxel or to the double (ACY1215, AZD6244) and triple (ACY1215, AZD6244, paclitaxel) combination of drugs for 48 h. For the double combination a 24 h pre-incubation with ACY1215 followed by a 24 co-incubation of ACY1215 and AZD6244 was also tested. At the end of treatment, floating and adherent cells were harvested for detection of apoptotic cells by Annexin V-binding assay (Immunostep, Salamanca, Spain). Cells were washed with cold PBS and re-suspended in binding buffer (10 mM HEPES-NaOH, pH 7.4, 2.5 mM CaCl_2_, and 140 mM NaCl, Immunostep). A fraction of 10^5^ cells was incubated in binding buffer at room temperature in the dark for 15 min with 5 μL of FITC-conjugated Annexin V and 10 μL of 2.5 μg/mL propidium iodide (Immunostep). Annexin V binding was detected by flow cytometry. At least 10^4^ events/sample were acquired and analyzed using specific software (CellQuestPro, Becton Dickinson).

### Western Blot Analysis

Western blot analysis was carried out as described (Corno et al., 2017). Briefly, samples were fractionated by SDS-PAGE and blotted on nitrocellulose membranes. Blots were pre-blocked in PBS containing 5% (w/v) dried no fat milk, and then incubated overnight at 4°C with the following antibodies: anti-phospho-Akt (Ser473), anti-Akt (BD Science, Franklin Lakes, NJ, United States), anti-phospho-ERK1/2 (Thr202/Tyr204, Thr185/Tyr187), anti-ERK1/2, anti-AR (Millipore, Burlington, MA, United States); anti-Hsp90 (ac-Lys294) (Novus, Centennial, Colorado, United States), anti-Hsp90 (Santa Cruz Biotechnology, Dallas, TX, United States), anti-acetylated alfa-tubulin (Sigma-Aldrich, Milan, Italy), anti-Bax and anti-FLIP_*L*_ (Sigma-Aldrich, Milan, Italy), anti-p53 (Dako, Santa Clara, CA, United States), anti-cleaved caspase-3 (Asp175) and anti-cleaved caspase-7 (Asp198) (Cell Signaling, Danvers, MA, United States). Anti-vinculin (Sigma-Aldrich, Milan, Italy), anti-β-tubulin (Abcam, Cambridge, United Kingdom) or anti-actin (Sigma) antibodies were used as control for loading. Antibody binding to blots was detected by chemo-luminescence (Amersham Biosciences, Cologno Monzese, Italy). Three independent experiments were performed.

### HDAC6 Loss of Function Studies

22Rv1 cells were plated in 6-well plates (25,000 cells/cm^2^) and 24 h later they were transfected using Opti-MEM transfection medium (Gibco by Life Technologies) and Lipofectamine 3000 (Thermo Fisher Scientific), with 10 nM of small interfering RNA (siRNA) to HDAC6 (SMARTpoolsiRNA, Dharmacon, Horizon Discovery Ltd, Cambridge, United Kingdom) or negative control siRNA (Silencer Select Negative Control #2 siRNA, Life Technologies). The transfection mix was added to complete medium for 24 h and then it was replaced with cell medium. Transfection efficiency was evaluated by quantitative Real-Time PCR (qRT-PCR) as indicated, 48 and 72 h after transfection start. Cells were harvested 48 h after transfection start and were re-seeded in 6-well plates at a density of 17,000 cells/cm^2^ for apoptosis evaluation by Annexin V-binding assay (Immunostep, Salamanca, Spain), performed after the treatment with AZD6244 for 24 h.

DU145 cells were plated in 6-well plates and 24 h later cells were transfected using Opti-MEM transfection medium and Lipofectamine RNAiMAX (Thermo Fisher Scientific) with 3 nM HDAC6 siRNA or negative control siRNA. Cells were incubated with transfection mix for 5 h and then the transfection medium was replaced with complete medium. Transfection efficiency was evaluated by qRT-PCR 72 h after transfection start. Cells were harvested 72 h after transfection start and were re-seeded in 12-well plates at a density of 10^4^ cells/cm^2^ for apoptosis evaluation by Annexin V-binding assay (Immunostep, Salamanca, Spain), performed after the treatment with AZD6244, paclitaxel or their combination (72 h).

### Quantitative Real Time PCR

RNA was isolated using the RNeasy Plus Mini Kit (Qiagen, Hilden, Germany). Reverse transcription was carried out using 1 μg RNA in the presence of RNAse inhibitors, using the High Capacity cDNA Reverse Transcription Kit according to manufacturer protocol (Applied Biosystems, Foster City, CA, United States). Gene expression was determined by quantitative real time PCR (qRT-PCR) using TaqMan assays (HDAC6, Hs00195869_m1; Applied Biosystems; DUSP1, Hs.PT.58.39287533.g; KLK2, Hs.PT.58.4099919.g; GAPDH, Hs.PT.39a.22214836; IDT). Technical triplicate reactions were carried out in 10 μl containing 2.5 μl cDNA, 5 μl master mix (TaqMan UniversalFast PCR Master Mix, Applied Biosystems), 0.5 μl of the specific assay. Reactions were performed using a 7900HT Fast Real-Time PCR System (Applied Biosystems) equipped with SDS (Sequence Detection Systems) 2.4 software (Applied Biosystems). Data analysis was performed with RQ manager software (Applied Biosystems). Relative levels of cDNA were determined as previously described (Corno et al., 2017), through the relative quantification (RQ) method. Untransfected or control cells were chosen as calibrator.

### Confocal Microscopy Analysis

One hundred thousand cells were seeded in 12-well plates containing circular coverslips slides. Twenty-four hour later, cells were exposed to drugs. Specifically, cells were pre-incubated with 3 μM ACY1215 for 24 h and then 30 or 100 μM AZD6244 was added for 24 h. Cells were then fixed in 3% paraformaldehyde (Merck, Darmstadt, Germany) in PBS for 15 min at room temperature and then permeabilized in 99.9% methanol for 1 min at room temperature. After washing in PBS, cells were incubated for 1 h in PBS containing 2% bovine serum albumin (BSA). The coverslips slides were incubated for 1 h at room temperature with the primary antibody against AR (1:100, Millipore, 06-680) diluted in PBS-2% BSA. The slides were then washed in PBS, and incubated for 1 h at room temperature with the secondary antibody conjugated with AlexaFluor488 (1:500, Molecular Probes, Thermo Fisher). Samples were counterstained with Hoechst 33342 for 2 min and mounted with Prolong Gold AntiFade Reagent (Thermo Fisher). Slides were left overnight to dry and images were collected.

The sample imaging was performed using a confocal laser scanning microscope Leica TCS SP8 X (Leica Microsystems GmbH, Mannheim, Germany). The fluorochromes were excited by a continuous wave 405 nm diode laser and a pulsed super continuum White Light Laser (412–470 nm; 1 nm tuning step size). The images were acquired in the scan format 512 × 512 pixel in a Z stack series (step size 0.5 μm) using a HC PL APO and 40X/1.30 CS2 oil immersion objective and a pinhole set to 1 Airy unit. The data were analyzed using the Leica LAS AF rel. 3.3 software (Leica Microsystems GmbH, Mannheim, Germany). The images were analyzed using “ImageJ” software (Abramoff et al., 2004). To evaluate fluorescence intensity, 10 different cells were analyzed from each picture. Fluorescent relative intensity of each cell was measured by drawing a region of interest (ROI) over cell perimeter; cytoplasm fluorescent intensity was obtained by subtracting the fluorescence of nuclei from the whole fluorescence.

### Statistical Analysis

Statistical analyses were performed using the GraphPad PrismTM software (GraphPad Software, San Diego, CA, United States). For comparison of IC_50_ values, ANOVA was used followed by Bonferroni’s multiple comparison tests and Mann Whitney test as indicated. Other comparisons were carried out using 2 sided Student’s *t* test.

## Results

### Sensitivity of Prostate Carcinoma Cells to Conventional and Targeted Antitumor Agents

Sensitivity of the prostate carcinoma DU145, PC3 and 22Rv1 cells to the MEK inhibitor AZD6244, the HDAC inhibitor ACY1215 and paclitaxel was assessed by growth-inhibition assays following 72 h drug exposure (Supplementary Table 1). AZD6244 presented the most marked anti-proliferative effect in DU145 cells (*P* < 0.05, by ANOVA – Bonferroni’s test). 22Rv1 cells displayed an intermediate cell sensitivity to AZD6244, the IC_50_ value being around 30 μM. PC3 cells were poorly sensitive to the MEK inhibitor with an IC_50_ of 80 μM. ACY1215 exhibited a comparable anti-proliferative effect in all the tested cell lines, with IC_50_ values in the micromolar range. Paclitaxel was more potent than targeted agents with IC_50_ values in the nanomolar range in the three cell lines.

### Analysis of the Interaction Between AZD6244 and ACY1215 in Prostate Carcinoma Cells

**FIGURE 1:**
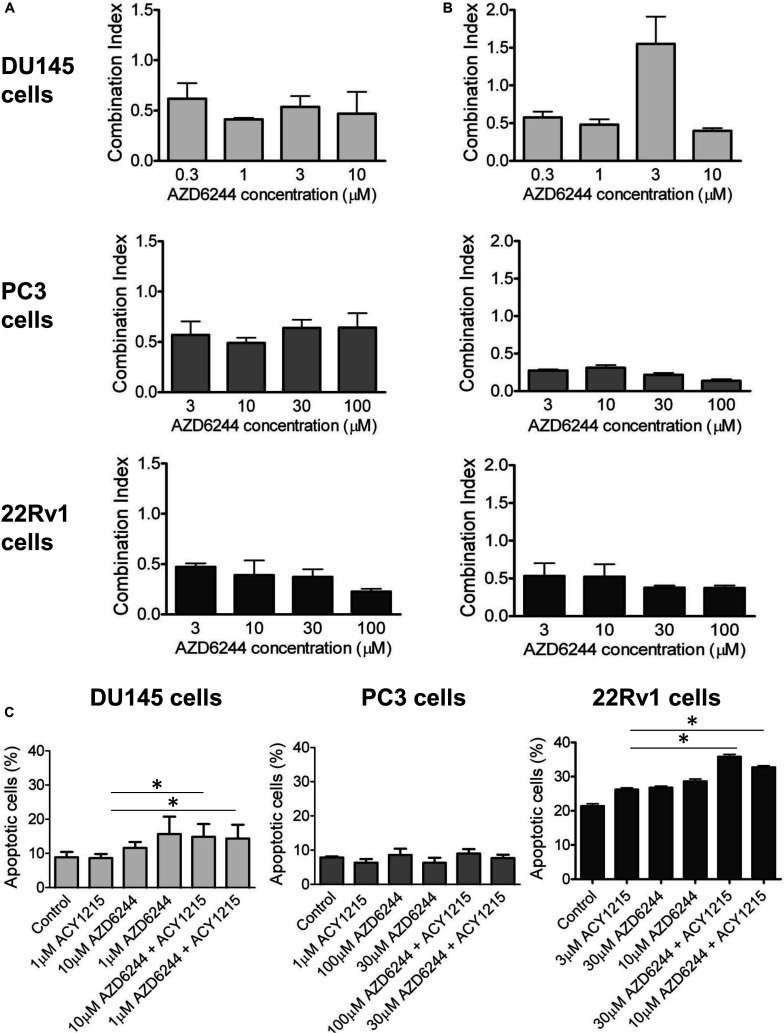
Analysis of the drug interaction and apoptotic response in prostate carcinoma cells exposed to the MEK inhibitor AZD6244 and to the HDAC inhibitor ACY1215. Cell sensitivity to increasing concentrations of selumetinib (AZD6244) and ricolinostat (ACY1215) or to their combination was assessed by growth inhibition assays in DU145, PC3, and 22Rv1 cells. Cells were exposed for 72 h to each drug alone or to the drug combination with 1 μM (for DU145, PC3 cells) or 3 μM (for 22Rv1 cells) ACY1215. Histograms of the mean of Combination Index values of at least 3 independent experiments are shown **(A)**. A 24 h pre-treatment with 1 μM (for DU145, PC3 cells) or 3 μM (for 22Rv1 cells) ACY1215, was followed by 48 h co-incubation with AZD6244. Histograms of the mean of Combination Index values of at least 3 independent experiments are shown **(B)**. Cells were exposed to single agents or to their combination according to a simultaneous schedule for DU145 cells and 24 h pre-treatment with ACY1215 followed by 24 h co-incubation with AZD6244 for PC3 and 22Rv1 cells, and harvested 48 h after treatment start for analysis of apoptosis by Annexin V binding assay **(C)**.

We observed a synergistic interaction in DU145, PC3 and 22Rv1 cells, as indicated by the CI values, using a simultaneous 72 h combination treatment with increasing concentrations of the MEK inhibitor and a sub-toxic concentration of the HDAC inhibitor (Figure 1A). The drug combination was particularly effective in 22Rv1 cells treated with 3 μM ACY1215, the CI values being in the range 0.2–0.4. When prostate carcinoma cells were pre-treated for 24 h with ACY1215, and then exposed to increasing concentrations of AZD6244 for additional 48 h, a synergistic interaction between the two small-molecule inhibitors was also found, as indicated by the CI values (Figure 1B). Under such experimental conditions, the most favorable drug interaction was observed in PC3 cells, with CI values in the range of 0.2–0.5 when using 1 μM ACY1215. In 22Rv1 cells, a synergistic interaction was evident upon exposure to 3 μM ACY1215 combined with various concentrations of AZD6244.

### Analysis of Apoptosis in Response to the Drug Combinations Between AZD6244 and ACY1215 in Prostate Carcinoma Cells

**FIGURE 2:**
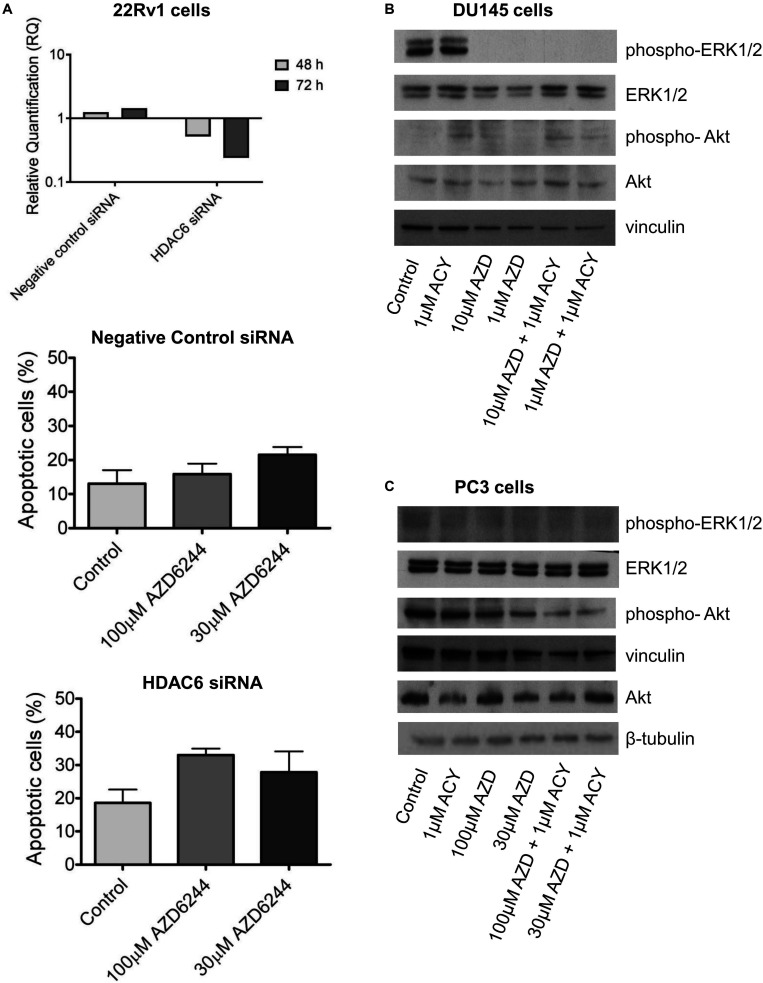
HDAC6 loss of function studies in 22Rv1 cells and analysis of target modulation in prostate carcinoma cells exposed to the combination of AZD6244 and ACY1215. Knockdown of HDAC6 by siRNA transfection in 22Rv1 cells. qRT-PCR analysis of HDAC6 mRNA levels at different times after transfection start; untrasfected cells were used as calibrator and GAPDH as housekeeping gene. Analysis of apoptosis in transfected cells exposed to MEK inhibitor AZD6244 for 24 h. At the end of treatment cells were harvested for analysis of apoptosis by Annexin V binding assay **(A)**. Western blot analysis **(B,C)** of possible targets was carried out in DU145 and β-tubulin **(B)** and PC3 **(C)** cells incubated with AZD6244 and ACY1215 or their combination, according the most favorable schedule. Control loading is shown by vinculin. The protein band intensity was quantified using ImageJ, normalized to that of the loading control and expressed relative to the level of control cells (set to 1). Normalized values corresponding to 1 μM ACY1215, 10 μM AZD6244, 1 μM AZD6244, 10 μM AZD6244 plus 1 μM ACY1215, 1 μM AZD6244 plus 1 μM ACY1215 were 1.30, 0.02, 0.04, 0.02, 0.003 for phospho-ERK1/2; 1.35, 1.34, 1.29, 2.66, 2.43 for ERK1/2; 6.91, 9.92, 5.88, 16.59, 4.55 for phospho-Akt; 1.89, 1.03, 2.52, 3.61, 2.20 for Akt, respectively **(B)**. Normalized values corresponding to 1 μM ACY1215, 100 μM AZD6244, 30 μM AZD6244, 100 μM AZD6244 plus 1 μM ACY1215, 30 μM AZD6244 plus 1 μM ACY1215 were 0.85, 0.78, 1.13, 1.59, 1.44 for phospho-ERK1/2; 0.93, 1.06, 1.41, 2.10, 2.22 for ERK1/2; 1.06, 1.03, 0.79, 0.73, 0.58 for phospho-Akt; 0.55, 0.76, 0.54, 0.52, 0.84 for Akt, respectively; p-Akt/Akt ratio was 1.92, 1.35, 1.46, 1.40, 0.69 **(C)**.

To determine whether the drug interaction resulted in an enhancement of apoptotic cell death, we performed flow-cytometric analysis of apoptotic cells by PI/Annexin V assay (Figure 1C). Apoptosis was determined at 48 h after drug exposure start using for each cell line the most favorable schedule observed in cell sensitivity assays. In DU145 cells, the simultaneous combined treatment of 1 μM ACY1215 with AZD6244 (10 and 1 μM) produced a slight increase of apoptosis with respect to the treatment with the single agents (*P* ≤ 0.05 by unpaired Student’s *t* test, Figure 1C), whereas in PC3 cells pre-treated with 1 μM ACY1215 for 24 h, there was no substantial increase of the percentage of apoptotic cells following the combined drug exposure independently of the AZD6244 concentration (Figure 1C) and no activation of caspases upon treatment (Supplementary Figure 1A). Marginal levels of apoptosis were found in DU145 cells exposed to the combination according to a pre-incubation schedule (Supplementary Figure 2). Exposure of DU145 cells to a relatively low concentration (1 μM) of the MEK inhibitor AZD6244 could induce *per se* a marked level of apoptosis (around 15%), similar to that induced by the tested combinations. Consistently, a modest activation of caspase 3 and caspase 7 was observed upon treatment (Supplementary Figure 1B). In 22Rv1 cells exhibiting a higher basal level of apoptosis (Figure 1C), a significant amount of apoptotic cells (around 30%) was detected upon exposure to 30 μM AZD6244 or to its combination with 3 μM ACY1215 (*P* < 0.05 by unpaired Student’s *t* test of 30 μM AZD6244 *versus* cells treated with the combination of 30 μM AZD6244 and 3 μM ACY1215). In these cells, higher levels of cleaved caspase 3 and caspase 7 were also observed upon combined treatment (Supplementary Figure 1C). In 22Rv1 cells, a marked amount of apoptotic death was found also upon knock-down of HDAC6 by siRNAs (Figure 2A).

### Modulation of Biochemical Targets Assayed by Western Blot Analysis

To examine the possible contribution of specific pathways to the effects observed in drug-combination studies, we investigated the modulation of factors implicated in cell proliferation and survival pathways by Western blot (Figures 2B,C).

The effect of AZD6244 and ACY1215 – both as single agents and in combination – was investigated in the PC3 and DU145 cells, according to the schedule treatment providing the most favorable drug interaction. In DU145 cells, a marked down-regulation of phospho-ERK1/2 was observed upon 48 h drug exposure to AZD6244 and its simultaneous combination with ACY1215. No effect on Akt phosphorylation was found in these cells (Figure 2B). Due to the loss of PTEN expression, Akt is constitutively phosphorylated/activated in PC3 cells (Shen and Abate-Shen, 2010). Drug treatment with AZD6244 (100 and 30 μM) and ACY1215 (1 μM) decreased Akt phosphorylation in PC3 cells (Figure 2C). A marked down-regulation of ERK1/2 phosphorylation upon treatments was observed in 22Rv1 cells, exhibiting high phospho-ERK1/2 levels (Supplementary Figure 3). Thus, in all cell lines, the efficacy of the combination was associated with inhibition of the constitutively deregulated survival pathways.

**FIGURE 3:**
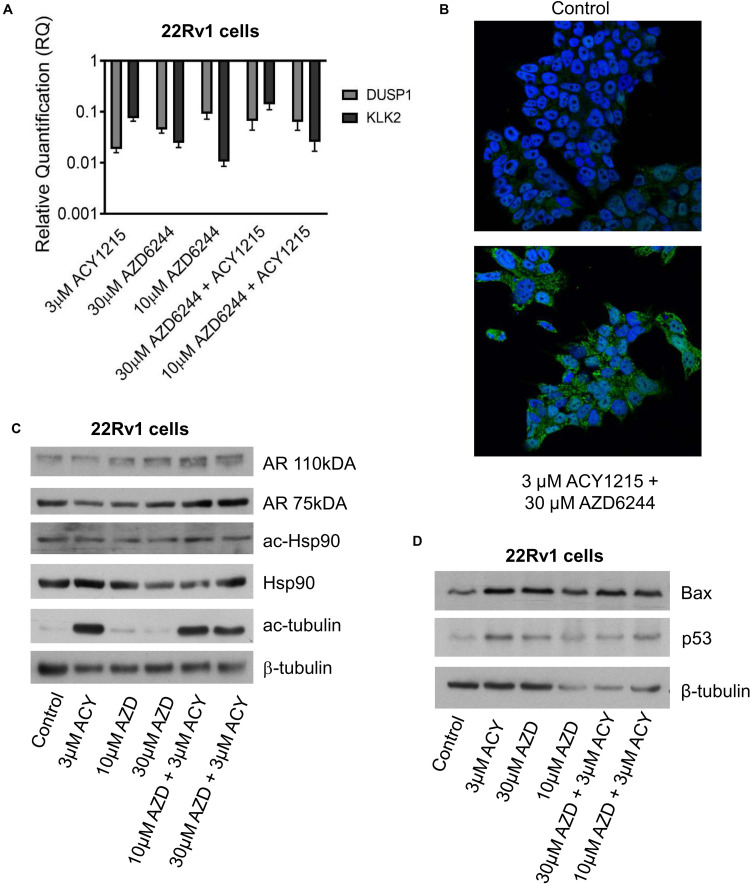
Analysis of target modulation in 22Rv1 cells exposed to the combination of AZD6244 and ACY1215. Quantitative RT-PCR of AR target genes (KLK2 and DUSP1) was carried out in 22Rv1 cells incubated with AZD6244 and ACY1215 or their combination, according 24 h pre-treatment with ACY1215 followed by 48 h co-incubation with AZD6244. Control cells were used as calibrator and GAPDH as housekeeping gene **(A)**. Representative image of immunofluorescence analysis by confocal microscopy of AR localization in 22Rv1 treated cells according to 24 h pre-treatment with ACY1215 followed by 24 h co-incubation with AZD6244 **(B)**. Western blot analysis of possible targets was carried out in 22Rv1 cells incubated with AZD6244 and ACY1215 or their combination, according 24 h pre-treatment with ACY1215 followed by 24 h co-incubation with AZD6244. Control loading is shown by β-tubulin. The protein band intensity was quantified using ImageJ, normalized to that of the loading control and expressed relative to the level of control cells (set to 1). Normalized values corresponding to 3 μM ACY1215, 10 μM AZD6244, 30 μM AZD6244, 10 μM AZD6244 plus 3 μM ACY1215, 30 μM AZD6244 plus 3 μM ACY1215 were 0.74, 1.11, 1.63, 1.96, 1.46 for AR 110 kDa; 0.58, 0.62, 0.96, 1.26, 1.25 for AR 75 kDa; 0.72, 0.67, 0.90, 1.10, 0.72 for ac-Hsp90; 1.08, 0.83, 0.57, 0.52, 0.83 for Hsp90; 21.61, 2.14, 2.36, 17.48, 15.19 for ac-tubulin, respectively **(C)**. Western blot analysis of apoptotic protein was carried out in 22Rv1 cells incubated with AZD6244 and ACY1215 or their combination, according 24 h pre-treatment with ACY1215 followed by 48 h co-incubation with AZD6244. Control loading is shown by β-tubulin. The protein band intensity was quantified using ImageJ, normalized to that of the loading control and expressed relative to the level of control cells (set to 1). Normalized values corresponding to 3 μM ACY1215, 30 μM AZD6244, 10 μM AZD6244, 30 μM AZD6244 plus 3 μM ACY1215, 10 μM AZD6244 plus 3 μM ACY1215 were 2.41, 2.46, 4.57, 5.71, 4.13 for Bax; 3.37, 2.29, 4.16, 4.09, 4.54 for p53, respectively **(D)**.

Since a favorable effect and apoptosis induction were obtained when combining ACY1215 and AZD6244 in 22Rv1 cells, that are characterized by marked phospho-ERK1/2 levels, by the expression of full length AR and of constitutively active AR variants (Tassinari et al., 2018), we focused our attention on the modulation of AR target genes by qRT-PCR analysis and investigated the levels of Kallicrein 2 (KLK2) and DUSP1 (Gregory et al., 1998; Vaarala et al., 2012, 466–472). A down-regulation was found for both genes (Figure 3A). This observation prompted us to assess by confocal microscopy whether an impairment of AR localization occurred upon treatment. AR localization was evidenced by indirect immunofluorescence using a secondary antibody conjugated to AlexaFluor488. AR was present in both cytoplasm and nuclei, but when 22Rv1 cells were exposed to the combination of compounds, a more brilliant fluorescent signal was detected in the cytoplasm (Figure 3B). Indeed, the cytoplasmic signal intensity was more marked in cells treated with the combination (mean intensity of 40% in treated cells versus 33% in control cells after normalization for cell area), suggesting a drug-induced delocalization of AR (Supplementary Table 2).

Since Hsp90 acetylation has been shown to result in disruption of the Hsp90-AR interaction and impaired nuclear AR localization can lead to proteasomal degradation (Seidel et al., 2016), we analyzed Hsp90 acetylation after treatment (Figure 3C). Under our experimental conditions, western blot analysis of Hsp90 – acetylated at the Lys294 residue – indicated no change in acetylation of the chaperone, with negligible modulation of total Hsp90. Acetylation of tubulin – a marker of HDAC6 inhibition – was observed in cells treated with ACY1215. Drug treatment resulted in increased Bax and p53 protein levels under all experimental conditions (Figure 3D).

### Analysis of the Interaction of AZD6244, ACY1215 and Paclitaxel in Prostate Carcinoma Cells

**FIGURE 4:**
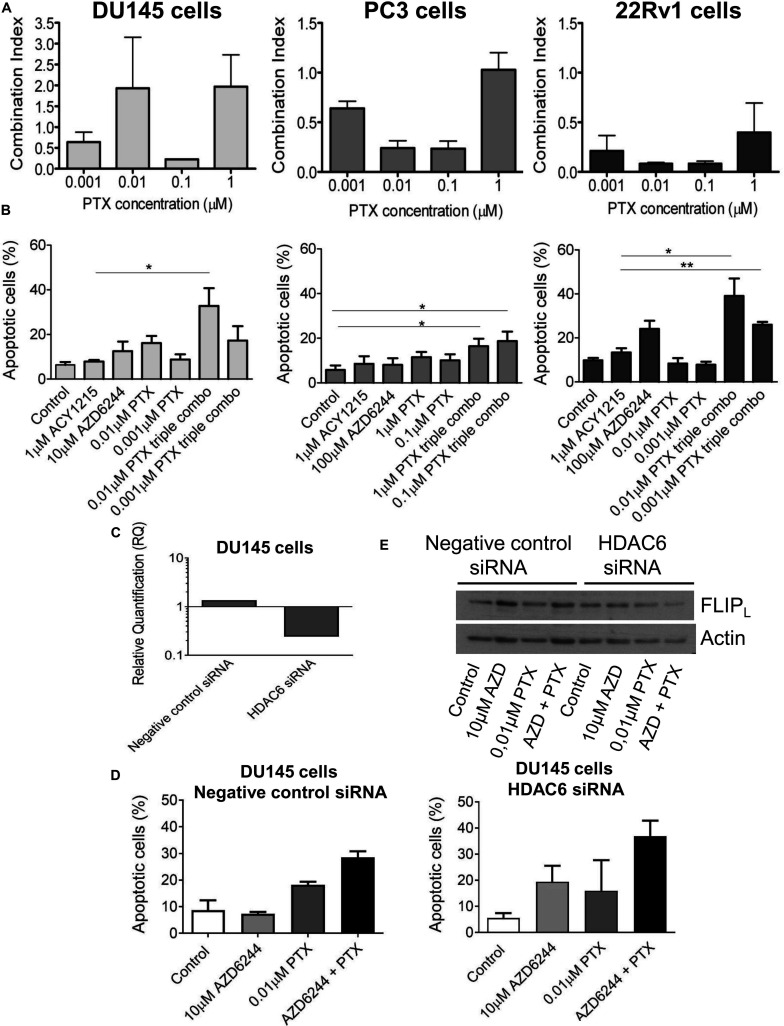
Analysis of the drug interaction and apoptotic response in prostate carcinoma cells simultaneously treated with the MEK inhibitor AZD6244, the HDAC inhibitor ACY1215 and paclitaxel. Cell sensitivity to increasing concentrations of selumetinib (AZD6244), ricolinostat (ACY1215) and paclitaxel (PTX) or to their combination was assessed by growth inhibition assays in DU145, PC3 and 22Rv1 cells. Cells were exposed for 72 h to each drug alone or to the drug combination with 1 μM ACY1215 (for all cell lines) and 10 μM AZD6244 (for DU145 cells) or 100 μM AZD6244 (for PC3 and 22Rv1 cells). Histograms of the mean of Combination Index (CI) values of at least 3 independent experiments are shown **(A)**. Cells were exposed to single agents or to their combination and harvested 48 h after treatment start for analysis of apoptosis by Annexin V binding assay; *P* < 0.05 by *t* test **(B)**. Knockdown of HDAC6 by siRNA transfection in DU145 cells. qRT-PCR analysis of HDAC6 mRNA levels at 72 h after transfection start; untrasfected cells were used as calibrator and GAPDH as housekeeping gene **(C)**. Analysis of apoptosis in transfected cells exposed to MEK inhibitor AZD6244 and PTX or to their combination (72 h). At the end of treatment cells were harvested for analysis of apoptosis by Annexin V binding assay **(D)**. Western blot was carried out in DU145 transfected cells incubated with AZD6244 and PTX or their combination (72 h). Control loading is shown by actin. The protein band intensity was quantified using ImageJ, normalized to that of the loading control and expressed relative to the level of control cells (set to 1). Normalized values of FLIP_*L*_ corresponding to 10 μM AZD6244, 0.01 μM PTX, 10 μM AZD6244 plus 0.01 μM PTX, were 1.52, 1.34, 1.19 for negative control siRNA; 1.15, 1.35, 0.63 for HDAC6 siRNA, respectively **(E)**.

Because taxanes are used in the clinical treatment of prostate cancer patients and are known to exhibit a pro-apoptotic effect, we examined whether PTX displayed a favorable interaction with the tested combination of targeted agents. We observed a favorable drug interaction, as indicated by the CI values, using a simultaneous 72 h combination treatment with increasing PTX concentrations and different concentrations of the MEK (10 μM for DU145 cells and 100 μM for PC3 and 22Rv1 cells, respectively) and HDAC inhibitors (i.e., 1 μM for all the three cell lines, that determined cell growth inhibition ≤30%) (Figure 4A). The drug combination was particularly effective and synergistic in 22Rv1 cells at all tested PTX concentrations, the CI values being in the range of 0.1–0.4.

A quantitative analysis of apoptosis by the Annexin V-binding assay indicated that PTX exposure of cells treated with the combination of the MEK and HDAC inhibitors produced marked levels of apoptosis in the cell lines, with a percentage of apoptotic cells around 40% both in DU145 and 22Rv1 cells (Figure 4B). Specifically, in DU145 cells a significantly increased apoptosis was evidenced when comparing 1 μM ACY1215-treated cells with cells exposed to the triple combination, including 10 μM AZD6244 and 0.01 μM PTX (*P* < 0.05 by unpaired Student’s *t* test). In DU145 cells, a marked down-regulation of HDAC6 mRNA levels was found upon molecular inhibition of HDAC6 by siRNAs (Figure 4C), with HDAC6 silencing resulting in enhanced apoptotic response following treatment with the MEK inhibitor AZD6244 compared to negative control cells (Figure 4D). This behavior was associated with a decrease of the levels of the anti-apoptotic protein and caspase-8 inhibitor FLIP_*L*_ in silenced cells exposed to the ACY1215-AZD6244 combination as compared to negative control transfected cells (Figure 4E).

The percentage of apoptotic cells was increased upon exposure to the triple combination in 22Rv1, DU145 and also in PC3 cells, in which marginal levels of apoptosis are usually detected (Figure 4B).

## Discussion

There has been a huge gain in knowledge on the genomic landscape of prostate cancer (Yap et al., 2016), but this has not been fully translated to the therapeutic setting. Targeted therapies have provided disappointing results when used as single agents in solid tumors, suggesting the importance of devising rational combinations of targeted drugs.

In the present study, we employed cell models of castration-resistant prostate cancer exhibiting activation of survival pathways, including the 22Rv1 cell line that displays a partial androgen-insensitive phenotype and represent an interesting model of clinical prostate carcinoma progression. We observed that the MEK inhibitor AZD6244 was less effective in inhibiting cell growth of PC3 than that of DU145 cells, an expected finding since PC3 cells carry PTEN gene deletion producing elevated Akt activation and Raf/MEK/Erk pathway suppression (Zhao et al., 2004; Perego et al., 2006). Conversely, DU145 PTEN-positive cells displaying constitutive activation of ERK1/2 (Perego et al., 2006; Carey et al., 2007) were found more sensitive to AZD6244 than PC3 cells.

A synergistic interaction between the MEK- and HDAC-inhibitors could be achieved in all cell lines, at most tested drug concentrations, according to a simultaneous schedule of treatment or when cells were exposed to ACY1215 before treatment with AZD6244. An optimal drug interaction was found in DU145 cells with a simultaneous schedule and in PC3 and 22Rv1 cells with HDAC6 inhibitor pre-incubation, as documented by CI values. This observation suggests a contribution of the molecular background to drug response. Such a background may also underlie the increased basal apoptosis observed in 22Rv1 cells. Moreover, the impact of the molecular features on drug response is also supported by the fact that apoptosis induction upon combined treatment did not parallel synergism. Indeed, PC3 cells were not susceptible to drug-induced apoptosis given that less than 10% of apoptotic cells were observed upon combined treatment. Differently, both DU145 and 22Rv1 cells exposed to drug combinations underwent drug-induced apoptosis, which was more marked in 22Rv1 than in DU145 as also supported by activation of caspases. In these three tumor cell models, a suppression of survival pathways, i.e., Akt in PC3 and ERK1/2 in DU145 and 22Rv1 cells, was evidenced. These findings, together with results from growth inhibition and apoptosis assays, indicate that suppression of survival pathways does not necessarily affects apoptosis induction. Although MAPK acts downstream of different pathways, MEK inhibition seems to have a more pronounced pro-apoptotic efficacy in prostate cancer models with ERK1/2 activation, like DU145 and 22Rv1 cells. A synergistic interaction in terms of proliferation inhibition may – however-still be considered a favorable effect. In addition, molecular targeting of HDAC6 which warrantees the unique inhibition of the enzyme in the absence of off-target effects appears to be a good strategy to increase cell killing, as shown in 22Rv1 cells. In these cells which express the full length AR and constitutively active AR variants (Tassinari et al., 2018), a down-regulation of AR target genes, i.e., KLK2 (Guerrico et al., 2017) and DUSP1 (Vaarala et al., 2012), was observed. This phenomenon was not associated with a down-regulation of AR protein levels, but with a delocalization of the receptor which tended to be more cytosolic upon treatment, as shown by confocal microscopy. KLK2 has been reported to be involved in the regulation of AR through the cooperation with ARA70, in a positive loop that leads to the trans-activation of the receptor itself. Thus, the reduced expression of KLK2 may contribute to decrease AR activation (Niu et al., 2008).

To examine additional strategies increasing apoptotic cell death in prostate cancer cells we combined the HDAC6 and MEK inhibitors with PTX. Under these conditions, apoptosis induction was achieved also in PC3 cells. HDAC6 knockdown in DU145 cells resulted in increased apoptotic death upon exposure to AZD6244 and PTX, with lower levels of FLIP_*L*_ as compared to cells transfected with the negative control siRNA treated with the same drug combination. Because the caspase-8 inhibitor c-FLIP_*L*_ blocks induction of apoptosis mediated by death receptors (Shirley and Micheau, 2013), it is likely that the extrinsic pathway contributes to cell death induction in this cell line. Of note, we previously reported that c-FLIP_*L*_ was constitutively up-regulated in PC3 cells (Perego et al., 2006) in keeping with low susceptibility to drug-induced apoptosis.

Prostate cancer remains an important cause of cancer-related death in men. In the present study, we provide evidence that favorable drug interactions can be achieved in castration-resistant *in vitro* models of prostate cancer. The occurrence of cell death appears to be dependent on the molecular background unless conventional cytotoxic agents are used in combination with targeted agents.

## Data Availability Statement

The raw data supporting the conclusions of this article will be made available by the authors, without undue reservation, to any qualified researcher.

## Author Contributions

PP, LG, and NZ conceived the study with the contribution of NA, ECi, and CC. PP, LG, and CC wrote the manuscript. CC, NC, and ECo carried out growth inhibition assays, western blots and qRT-PCR analyses. ECi performed cytofluorimetric analyses of apoptosis. NA carried out confocal microscopy analysis. All authors contributed to manuscript revision and read and approved the submitted version.

## Conflict of Interest

The authors declare that the research was conducted in the absence of any commercial or financial relationships that could be construed as a potential conflict of interest.

## Abbreviations

AR: androgen receptor
DUSP1: dual specifity phosphatase 1
FLIP_*L*_: FLICE-like Inhibitory protein –long
HDAC: histone deacetylases
HDACi: HDAC inhibitor
Hsp90: heat shock protein 90
KLK2: Kallicrein 2
MAPK: mitogen activated protein kinase
PTX: paclitaxel.
