# Supplementary material for: Synergistic Interaction of Histone Deacetylase 6- and MEK-Inhibitors in Castration-Resistant Prostate Cancer Cells
Source: Front Cell Dev Biol. 2020 Jul 10;8:610. doi: 10.3389/fcell.2020.00610 (PMC7365948; doi:10.3389/fcell.2020.00610)
Supplement: Supplementary file 1 [file Presentation_1.pdf]

Supplementary Material

1 Supplementary Figures and Tables

1.1 Supplementary Table

Supplementary Table 1. Sensitivity of prostate carcinoma cells to selumetinib (AZD6244), ricolinostat (ACY1215) and paclitaxel (PTX).

		IC_{50} [μM][a]	
**Cell line**	**AZD6244**	**ACY1215**	**PTX**
**DU145**	3.1 ± 2.0	3.3 ± 2.0	0.025 ± 0.013
**PC3**	80.2 ± 23	4.3 ± 2.2	0.0080 ± 0.0008
**22Rv1**	32.7 ± 14.3	1.62 ± 0.4	0.019 ±0.0045

[a] Cell sensitivity was assessed by cell growth inhibition assay. Cells were exposed for 72 h to each drug. IC_{50}, drug concentration inhibiting cell growth by 50%. Values are the mean (± SD, standard deviation) of at least three independent experiments. Comparison of AZD6244 IC_{50} values by ANOVA followed by Bonferroni's Multiple Comparison Test: $P < 0.05$. Comparison of targeted drug IC_{50} values *versus* paclitaxel IC_{50} values by Mann Whitney test: $P < 0.0001$.

Supplementary Table 2. Immunofluorescence staining analysis of androgen receptor on 22Rv1 cell line.

	RI tot (±SD)	RI cyt (±SD)	%
**Control**	29.8 (±5.9)	9.9 (±3.1)	33.2
**3µM ACY1215**	32.8 (±6.8)	12.4 (±4.9)	37.8
**10µM AZD6244**	42.6 (±11.6)	14.6 (±7.4)	34.2
**30µM AZD6244**	48.1 (±8.3)	14.9 (±6.5)	31.0
**ACY1215 + 10µM AZD6244**	43.7 (±8.2)	17.4 (±4.2)	39.8
**ACY1215 + 30µM AZD6244**	63.2 (±7.4)	25.3 (±3.8)	40.0

Cells were exposed to ACY1215 for 24 h, followed by 24 h co-incubation with AZD6244. At the end of the treatment cells were fixed, permeabilized and incubated with the primary antibody against AR and the secondary antibody conjugated with AlexaFluor488. RI = relative intensity, obtained from the ratio between total intensity and analyzed area. RI Values are the mean (±SD, standard deviation) of the RI obatined from 10 cells.

1.2 Supplementary Figures

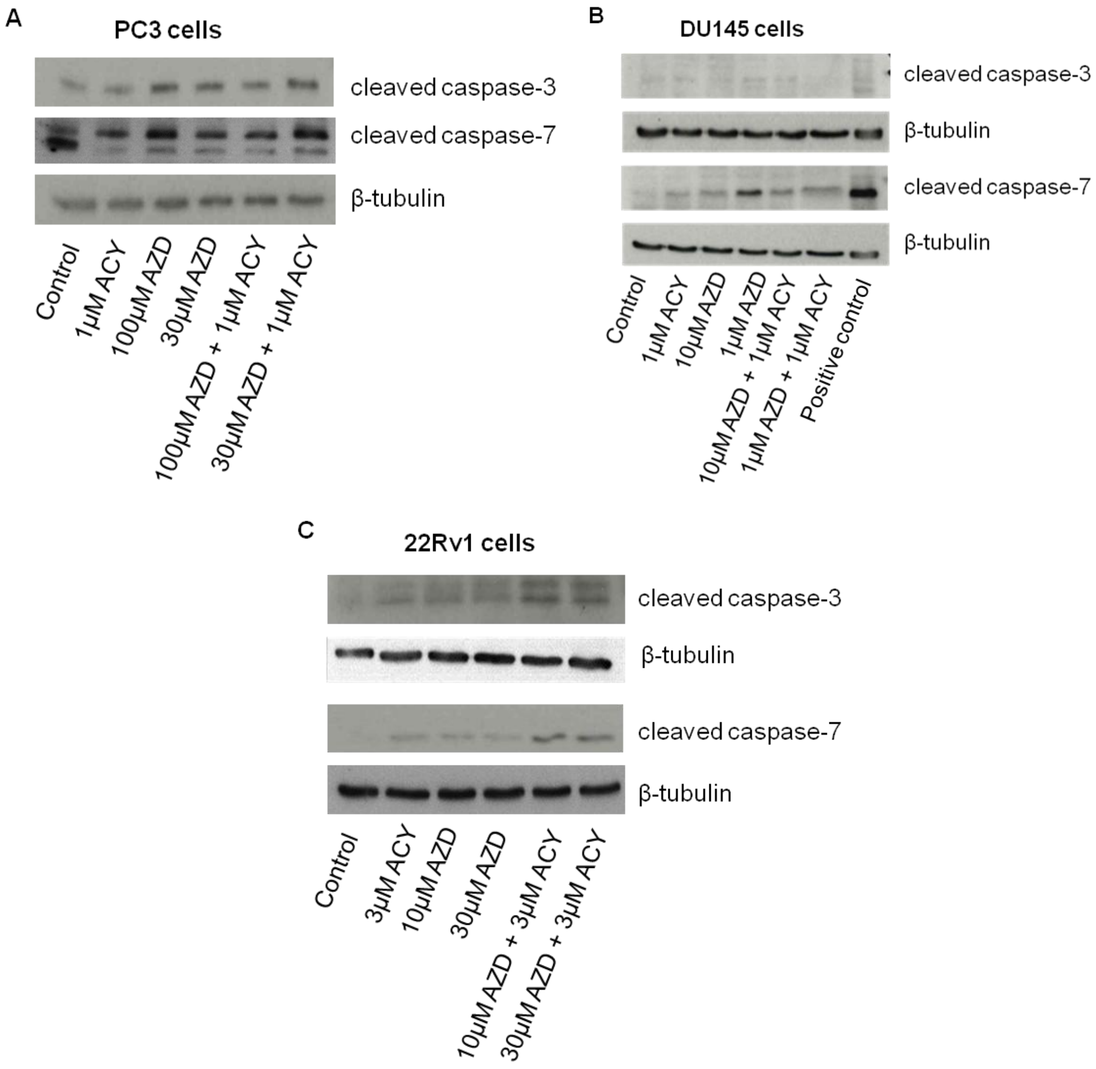

Supplementary Figure 1. Analysis of caspase 3/7 modulation in prostate carcinoma cells exposed to the combination of AZD6244 and ACY1215. Western blot analysis of caspase modulation was carried out in PC3 (A), DU145 (B) and 22Rv1 (C) cells incubated with AZD6244 and ACY1215 or their combination for 48 h (A-C) or 72 h (B), according the most favorable schedule. Control loading is shown by β-tubulin. The protein band intensity was quantified using ImageJ, normalized to that of the loading control and expressed relative to the level of control cells (set to 1). Normalized values corresponding to 1µM ACY1215, 100µM AZD6244, 30µM AZD6244, 100µM AZD6244 plus 1µM ACY1215, 30µM AZD6244 plus 1µM ACY1215 were for 1.08, 1.64, 1.64, 1.23, 2.37 caspase-3; 0.07, 0.17, 0.12, 0.09, 0.17 for caspase-7, respectively (A). Normalized values corresponding to 1µM ACY1215, 10µM AZD6244, 1µM AZD6244, 10µM AZD6244 plus 1µM ACY1215, 1µM AZD6244 plus 1µM ACY1215 were for 0.74, 0.25, 1.16, 0.98, 0.42 caspase-3; 5.82, 7.53, 17.42, 9.27, 11.44 for caspase-7, respectively; positive control consists of a lysate from 22Rv1 cells treated with 10µM AZD6244 plus 3µM ACY1215 harvested 48 h after exposure (B). Normalized values corresponding to 3µM ACY1215, 10µM AZD6244, 30µM AZD6244, 10µM AZD6244 plus 3µM ACY1215, 30µM AZD6244 plus 3µM ACY1215 were 3.13, 4.29, 3.62, 7.17, 5.52 for caspase-3; 5.09, 3.95, 2.91, 11.49, 12.42 for caspase-7, respectively (C).

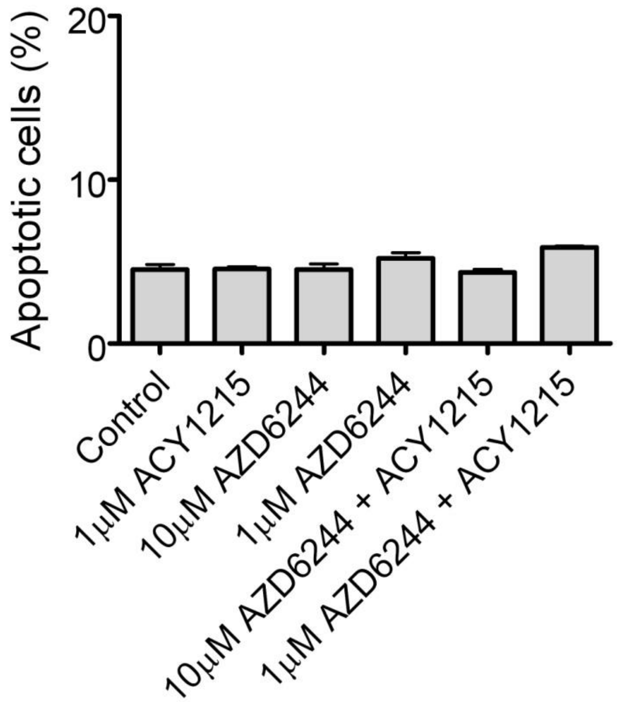

Supplementary Figure 2. Analysis of apoptotic response in DU145 cells exposed to the MEK inhibitor AZD6244 and to the HDAC inhibitor ACY1215. DU145 cells were exposed to single agents or to their combination, according to a 24 h pre-treatment with 1 µM ACY1215 followed by co-incubation with 10µM or 1µM AZD6244. Cells were harvested 48 h after treatment start for analysis of apoptosis by Annexin V-binding assay.

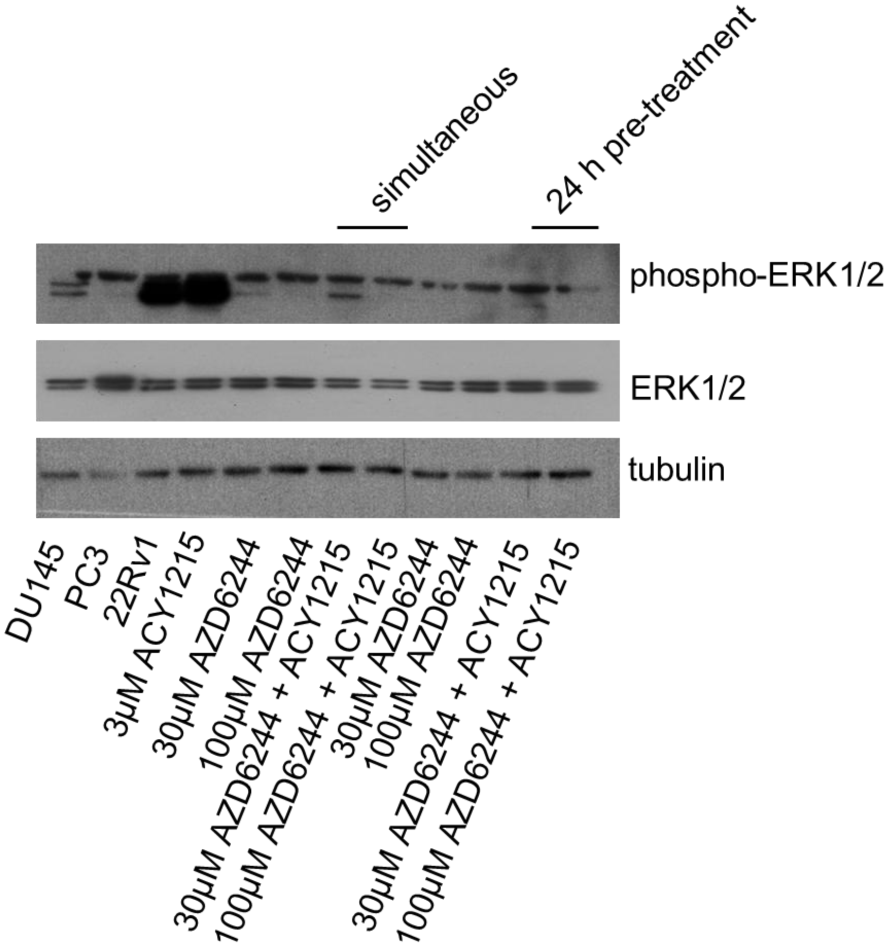

Supplementary Figure 3. Analysis of survival pathway modulation in 22Rv1 cells exposed to the combination of AZD6244 and ACY1215. Western blot analysis was carried out in 22Rv1 cells incubated with AZD6244 and ACY1215 or their combination, according simultaneous and 24 h pre-treatment with ACY1215 schedule. Control loading is shown by β-tubulin. The protein band intensity was quantified using ImageJ, normalized to that of the loading control and expressed relative to the level of control cells (set to 1). Normalized values corresponding to 3µM ACY1215, 30µM AZD6244, 100µM AZD6244, 30µM AZD6244 plus 3µM ACY1215, 100µM AZD6244 plus 3µM ACY1215 were 0.84, 0.34, 0.18, 0.22, 0.16 for phospho-ERK1/2; 0.87, 0.77, 0.57, 0.65, 0.68 for ERK1/2, simultaneous treatment; 0.84, 0.15, 0.19, 0.16, 0.07 for phospho-ERK1/2; 0.87, 1.32, 1.28, 1.07, 0.98 for ERK1/2, 24 h pre-treatment schedule, respectively.
